# It’s prime time for multiplexed prime editing

**DOI:** 10.1016/j.xgen.2025.100852

**Published:** 2025-04-09

**Authors:** Ke Wu, Francisco J. Sánchez-Rivera

**Affiliations:** 1 David H. Koch Institute for Integrative Cancer Research, Massachusetts Institute of Technology, Cambridge, MA 02142, USA; 2 Department of Biology, Massachusetts Institute of Technology, Cambridge, MA 02142, USA

## Abstract

Prime editing screens allow precise and scalable studies of genetic variants in their native genomic context but are limited by variable editing efficiency. In this issue of *Cell Genomics*, Herger, Kajba, et al.^1^ overcome these challenges by optimizing and applying prime editing screens to investigate variants in *SMARCB1* and *MLH1*.

## Teaser

Prime editing screens allow precise and scalable studies of genetic variants in their native genomic context but are limited by variable editing efficiency. In this issue of *Cell Genomics*, Herger, Kajba, et al. overcome these challenges by optimizing and applying prime editing screens to investigate variants in *SMARCB1* and *MLH1*.

## Main text

Large-scale sequencing studies have cataloged millions of disease-associated mutations; however, the pathogenicity of many of these variants and their functional roles in disease development remain largely unknown. Multiplexed assays of variant effects (MAVEs) have emerged as powerful tools for evaluating the functional consequences of thousands of genetic variants simultaneously.^2^ However, these studies often rely on exogenous, supraphysiological expression of variants, limiting our ability to interrogate genetic variants in their endogenous context. In contrast, precision genome editing technologies, such as base and prime editing (PE), enable the installation of a diverse range of genetic variants in their endogenous context with single-nucleotide resolution. Base editors can engineer edits genome-wide with high efficiency, but their utility is largely restricted to specific base-pair substitutions (C⋅G → T⋅A or A⋅T → G⋅C transitions), and they may produce unintended off-target effects from bystander editing. Prime editors, on the other hand, can precisely install all possible base-pair substitutions, as well as small insertions and deletions, but are often limited by lower editing efficiency. Recent studies have shown that optimizing PE systems, such as by incorporating engineered PE guide RNAs (epegRNAs)^3^ and dominant-negative MLH1 (MLH1dn),^4^^,^^5^ can improve desired editing outcomes. Despite these advancements, it remains unclear whether these enhanced PE systems can be applied effectively to characterize the functional impact of disease-associated variants in pooled screens.

**Figure 1:**
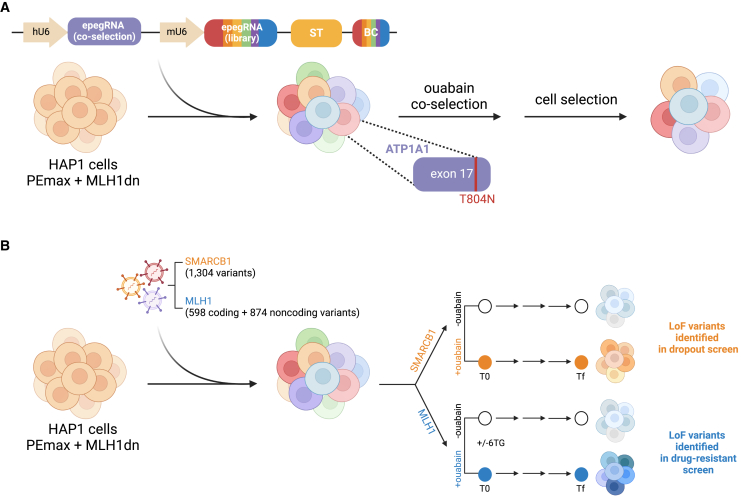
Optimized prime editing screening approach to assess the impact of variants in essential genes and to identify drug-resistant mutations (A) Herger, Kajba, et al. improved prime editing efficiency by incorporating stable PEmax and dominant-negative MLH1 (MLH1dn) and optimized epegRNA design, “surrogate target” (ST) sequences to assess pegRNA activity, and a co-selection system to enrich edited cells by employing a second pegRNA to target the essential *ATP1A1* gene, whose T804N mutation confers resistance to the Na^+^/K^+^-ATPase inhibitor ouabain. (B) Two proof-of-concept mutagenesis screens identified the depletion of LoF variants in the essential *SMARCB1* gene via negative selection, as well as the enrichment of LoF-resistant variants in MLH1 via positive selection with 6-thioguanine (6TG). T0, initial time point; Tf, final time point.

In this issue of *Cell Genomics*, Herger, Kajba, et al. developed and applied an optimized PE screening platform in haploid human cells (HAP1).^1^ To demonstrate this platform’s robustness and efficiency, they conducted two proof-of-concept mutagenesis screens targeting loss-of-function (LoF) variants in the essential gene *SMARCB1* and the non-essential gene *MLH1*, utilizing negative and positive selections, respectively. To overcome the low editing efficiency of PE at baseline, the authors adopted several well-established optimizations, including improved epegRNA design^6^ and stable expression of the PEmax prime editor linked to a MLH1dn.^4^ They also integrated multiple strategies to enhance variant installation, including (1) optimal pegRNA scaffolds, (2) PAM-disruptive silent mutations, (3) a sensor-like “surrogate target” (ST) that links pegRNA editing activity to its cognate target sequence, and (4) enrichment of edited cells via ouabain-mediated co-selection. This powerful co-selection employs a second pegRNA targeting a specific mutation in *ATP1A1*, a HAP1-essential gene, which confers resistance against the Na^+^/K^+^-ATPase inhibitor ouabain (Figure 1A).

As a proof of concept, the authors deployed their platform in HAP1 cells to identify LoF variants in *SMARCB1*, an essential gene encoding a core subunit of the BAF chromatin remodeling complex and whose LoF mutations are expected to be depleted in a proliferation screen (Figure 1B). They achieved significantly improved editing efficiencies by designing and screening a tiling library of 10,954 pegRNAs targeting all possible single-nucleotide variants (SNVs), multiple-nucleotide variants (MNVs) encoding nonsense mutations, and 3-bp deletions across two regions of *SMARCB1*. Notably, 33% of pegRNAs showed over 75% editing efficiency at the ST locus in the presence of ouabain co-selection. To further investigate their system, they sequenced *SMARCB1* amplicons and observed a good correlation between editing rates at the ST and the endogenous target (ET) sites. These findings are consistent with recent studies showing that ST-like elements can serve as robust empirical proxies for ET editing by precision genome editors.^6^^,^^7^ After applying a stringent threshold to select variants with >75% ST editing, the authors identified 12 significantly depleted variants and validated the top hits as being strongly selected against in a competition assay.

To expand their platform to a broader genomic context, the authors designed a similar mutagenesis screen targeting both coding and noncoding regions of the *MLH1* gene, a tumor suppressor involved in DNA mismatch repair (MMR). Previous studies have shown that loss of MMR function in HAP1 cells leads to partial 6-thioguanine (6TG) resistance, enabling the use of 6TG for robust positive selection of candidate LoF variants in *MLH1*^8^ (Figure 1B). Similar to the *SMARCB1* screen, their results showed that ouabain co-selection allows significant enrichment of edited cells with higher ST editing. More importantly, the authors validated their platform’s accuracy by assigning each variant a function score based on their enrichment/depletion in the context of 6TG selection. These function scores were highly correlated with pre-labeled variant classifications reported in ClinVar (i.e., benign, pathogenic, uncertain). These results substantiated that multiplexed PE screens can not only validate known variants but also uncover and functionally interrogate previously unidentified pathogenic variants, including many variants of uncertainty.

The work presented by Herger, Kajba, et al. marks a significant technological advancement in the field of precision genome editing. Their optimized PE platform enhances the utility of these technologies for systematically interrogating disease-associated genetic variants. By enabling the precise and versatile installation of genetic variants in their native context, multiplexed PE screens offer powerful tools to explore the mechanistic links between genotype and phenotype. There is still much work left to do in the field. One limitation of this study, as noted by the authors, is that their pegRNA scoring system tends to enrich variants with strong signal-to-noise ratios indicated by active pegRNAs. This could lead to the exclusion of false negatives, whose variant effects may be less pronounced due to their low pegRNA activity. To address this issue, further refinements incorporating pegRNA strength, ST editing efficiency, and dynamic co-selection strategies could improve the precision and sensitivity of variant scoring. Looking beyond the scope of this study, combining saturation mutagenesis of clinically relevant oncogenes, tumor-suppressor genes, and other classes of disease-relevant genes with diverse drugs and therapeutic modalities will provide valuable insights into mechanisms of therapeutic resistance and help identify *bona fide* drug-sensitive targets. Such approaches could ultimately drive the discovery of novel therapeutic treatments for cancer and other human diseases, bringing us closer to the ultimate goal of leveraging genome editing technologies to improve human health.

## Acknowledgments

Work in the Sánchez-Rivera laboratory is supported by the Howard Hughes Medical Institute (Hanna Gray Fellowship, GT15656), the V Foundation for Cancer Research (V2022-028), NCI Cancer Center support grant P30-CA014051, the Virginia and D.K. Ludwig Fund for Cancer Research, Koch Institute Frontier Research Program, the Casey and Family Foundation Cancer Research Fund, the Michael (1957) and Inara Erdei Fund, the MIT Research Support Committee, the Upstage Lung Cancer Foundation, and a Traditional Project Award from the Bridge Project, a partnership between the Koch Institute for Integrative Cancer Research at MIT and the Dana-Farber/Harvard Cancer Center. Figure 1 was created in BioRender.

## Declaration of interests

F.J.S.R. has been a consultant for Repare Therapeutics and Ono Pharma.
